# Production of graphene nanoplate/polyetheretherketone composites by semi-industrial melt-compounding

**DOI:** 10.1016/j.heliyon.2020.e03740

**Published:** 2020-04-08

**Authors:** A. Alvaredo-Atienza, Juan P. Fernández-Blázquez, P. Castell, R. Guzman de Villoria

**Affiliations:** a IMDEA Materials Institute, C/ Eric Kandel 2, 28906 Getafe, Madrid, Spain; b FIDAMC, Foundation for the Research, Development and Application of Composite Materials, Avda. Rita Levi Montalcini 29, Tecnogetafe, 28906 Getafe, Madrid, Spain; c Fundación AITIIP, Pol. Ind. Empresarium, C/ Romero 12, 50720 Zaragoza, Spain; d Department of Mechanical Engineering, University of Salamanca, Campus Viriato, Avenida Requejo, 33, 49022 Zamora, Spain

**Keywords:** Materials science, Nanotechnology, Aerospace engineering, Nanomaterials, PEEK, Graphene, Composites, Characterisation, Thermoplastic

## Abstract

Current studies on nanocomposites have focused on their multifunctional properties and their industrial production. In this work, polyetheretherketone (PEEK)/graphene nanoplate (GNP) composites were produced by a direct semi-industrial process. Different percentages of untreated GNP (1, 5, and 10 wt.%) were added to PEEK by employing melt-compounding followed by injection-moulding. Despite the semi-industrial approach used, the modulus, strength, and Poisson coefficient of the nanocomposites (1 and 5 wt.%) were not significantly affected by the addition of GNP. However, there was a slight decrease in the strength at 10 wt.% GNP. Our study also shows that the thermal conductivities of PEEK/GNP composites are up to 2.5 times higher than that of pure PEEK.

## 1 Introduction

Polyetheretherketone (PEEK) is a high-performance, semi-crystalline thermoplastic polymer that is used in the aeronautics, medical, and chemical industries as it has excellent mechanical properties including good thermal stability and chemical inertness [1, 2, 3]. However, because their relatively low glass transition temperature (≈140 °C), PEEK polymers are not recommended for structural applications in high-temperature environments [4].

Consequently, over the last decade, investigations have been conducted to improve the thermal properties of PEEK through the incorporation of carbon nanofillers such as carbon nanotubes, graphene, and graphene-based materials such as graphene nanoplates (GNPs) (also referred to as nanoplatelets) or graphene oxides [1, 5, 6, 7, 8, 9, 10, 11].

Considering the high cost of graphene, GNPs, which are formed from several stacked graphene layers bound to each other by Van der Waals forces, are an interesting option for large-scale production [12] of nanocomposite materials. High thermal conductivity (5000 W/mK) [13], high thermal stability in the range of 360–500 °C [14], and elastic modulus (≈1 TPa) [13] of GNPs are among the highest of the available large-scale carbon nanofillers [15, 16, 17].

The mechanical and thermal properties of a nanocomposite depend on the dispersion of the filler, its size distribution, and the interface between the matrix and the nano-reinforcements [18, 19, 20, 21]. Unfortunately, graphene, like most nanofillers, tends to form agglomerates [18]; dispersion and distribution remains a major problem for the effective reinforcement of graphene. The dispersion of graphene in PEEK is challenging because of its strong solvent resistance, high viscosity, and the high processing temperature of the PEEK matrix [6, 21].

Relative to other organic/inorganic filler nanocomposites with a PEEK matrix, there are few studies about PEEK/GNP composites available in the literature. In these studies, PEEK/GNP nanocomposites were prepared by two different methods: solution intercalation (with or without a compatibilising agent) [4, 5, 21] and melt-intercalation [6, 22, 23]. Solution intercalation is based on a solvent system where the polymer is soluble; this helps the reinforcement dispersion, whereas in melt-intercalation, the molten polymer penetrates the graphene layers of the agglomerates, thus avoiding re-agglomeration [24]. Of these two methods, solution intercalation gave the best results in terms of thermal conductivity (0.35 W/mK for 1 wt.% graphene oxide mixed by solution intercalation [5] versus 0.44 W/mK for 10 wt.% of GNP mixed by melt-intercalation [22]). Solution intercalation also resulted in better mechanical strength. For instance, increase in Young's modulus by 38% or tensile strength by 10% was obtained for solution intercalation [21], but for melt intercalation the increase in modulus was ≈30% with no change to or a slight decrease in tensile strength [22, 23].

Despite the inferior mechanical properties obtained by melt-compounding, the process is still preferred for industrial applications. The absence of solvents, easy-to-scale method, ready availability of industrial equipment among other qualities makes this technique a preferred choice for nanocomposite production [21, 24], especially when the aim is to improve the multifunctional properties of the materials without diminishing their mechanical properties.

In this work, PEEK/GNP composites were produced by a semi-industrial, two-step process of melt compounding followed by injection moulding. We studied the effects of GNPs (1, 5, and 10 wt.%) on the mechanical and thermal properties of the as-produced PEEK matrix. The fracture surfaces and the dispersion of graphene within the matrix of samples were analysed by scanning electron microscopy (SEM).

## 2 Materials and methods

### 2.1 Materials

PEEK 90P grade was provided in pellets by Victrex plc, UK, with glass transition temperature = 143 °C, melting temperature = 345 °C, density at 25 °C = 1.30 g/cm^3^, which were dried at 150 °C prior to be used for production of nanocomposites. Pristine graphene nanoplates (avan-GRAPHENE) were provided in powder form by Avanzare Innovación Tecnológica, SL (Spain). SEM analysis of the received graphene was performed by using a Helios Nano Lab 600i scanning electron microscope.

### 2.2 Nanocomposite preparation by extrusion moulding

PEEK nanocomposites were melt-blended using an extrusion-compounding machine, Coperion ZSK 26. This machine is equipped with a 26-mm diameter co-rotating twin-screw and two Brabender gravimetric feeders. Two different temperatures were set for this process: 330 °C for the extruder and 360 °C for the nozzle. A high shear rate screw profile, which was specifically designed for this process, was used to ensure proper dispersion. The rotor speed set to process all the nanocomposites was the same, 250 rpm. Two feeders were required for this process: one for feeding PEEK at a speed of 10 kg/h and a lateral one for feeding graphene. Graphene was fed in order to achieve a final concentration of the nanocomposites of 1, 5, and 10 wt.% raw GNP. The diameter of the die used for this process is 2 mm. Once the molten material was extruded through the die, it was quenched in a water bath at room temperature, then dried and cut into small pellets. Pure PEEK was also processed under the same conditions in order to use it as reference material.

### 2.3 Specimen preparation by injection moulding

Dog-bone shaped specimens (type V) for tensile tests, under ASTM D638-02a (Table 1), and specimens for flexural tests (79 mm × 10 mm × 4 mm), under ASTM D790-02, were injected in a mould made of tool steel (1.2790) at a constant temperature of 180 °C. The pellets were dried at 150 °C for 3 h prior to processing. The specimens were produced by injection moulding through a JSW 85 EL II injection machine with a 35-mm diameter reciprocating screw at a screw speed of 120 rpm. The dosage used in each specimen was 20 mm with an injection time of 0.36 s, the compaction pressure was 1000 bar, and the cooling time was 3 s. Pure PEEK and the specimens that contained different loadings of GNPs were prepared by following this protocol. At least 15 samples were produced for each composition.

**Table 1:** Dimensions of dog-bone tensile samples tested in accordance with standard ASTM D638-02a.

Symbol	Description	Dimension (mm)
T	Thickness	2
L0	Length overall	74.3
W	Width of narrow section	5
W0	Width overall	9
D	Distance between grips	37.4

### 2.4 Density measurement and morphological analysis of the samples

The density of the injection-moulded specimens with different percentages of graphene were measured in accordance with ASTM D792-13 standard. At least four cubic samples for each level of GNP content were produced and weighed in air and then in distilled water at room temperature (≈23 °C). The theoretical density was also calculated by applying the rule of mixtures with assumed densities of 1.3 g/cm^3^ for PEEK-90G and 2.2 g/cm^3^ for GNPs [25], and were analysed relative to the measured densities. Dispersion of graphene within the PEEK matrix in the injected samples was analysed by SEM (Helios NanoLab 600i, 2 KeV and 0.17 mA). The specimens were cooled in liquid nitrogen and immediately broken by using a razor blade hit with a hammer before a thin layer of gold (3 nm) was sputter-coated onto the surfaces.

### 2.5 Differential Scanning Calorimetry

Differential Scanning Calorimetry (DSC; Q200, TA instruments) was used to obtain information about the thermal properties of the nanocomposites. The samples (5–10 mg) of PEEK with different percentages of graphene (1, 5, and 10 wt.%) were heated from 20 °C to 400 °C at 10 °C/min and held at 400 °C for 5 min to remove the thermal history of the samples. Then the samples were cooled to 20 °C at 10 °C/min, held for 0.5 min and heated again to 400 °C at 10 °C/min. The DSC samples were cut from the bulk of the injection specimen (dog-bone) with the help of a hammer and a knife. One test per sample was carried out.

### 2.6 Mechanical characterisation

The characterisation of the flexural properties (flexural modulus, strength, and strain) was carried out in accordance with ASTM D790-02 in a three-point bending configuration with a distance between supports of 60 mm (Instron 338, 2KN load cell, 1.6 mm/min, 23 ± 2 °C). At least 5 specimens (79 mm × 10 mm × 4 mm) were measured for each composite.

Tensile tests were carried out under ambient conditions with an Instron 3384 by using a load cell of 10 kN. Tests were carried out in accordance with ASTM D638-02a by using type V specimens at 1 mm/min of crosshead speed (Table 1). The axial and transverse displacement (ε_1_ and ε_2_) of each specimen during the test was performed by digital image correlation (DIC). One side of every ‘dog bone’ specimen was painted white and then carefully and lightly sprayed with black paint to get the random speckle pattern required for DIC analysis. The images (36 mm × 4.5 mm) were taken every 2 s during the test [26, 27]. Finally, the images were evaluated by using Vic-2D 2009 DIC software (VicSNAP, Correlation Solutions Inc., Columbia, SC, USA). The fracture surfaces of the tested samples were also studied by SEM after they were sputtered with a 3-nm layer of gold (SEM Helios NanoLab 600i, 2KeV and 0.17mA).

### 2.7 Thermal conductivity

Thermal conductivity of PEEK/GNP composites was carried out by Hot Disk TPS (Hot Disk AB, Gothenburg, Sweden) in accordance with ISO 22007-2:2008 standard. Two identical samples (30 mm × 10 mm × 4 mm) were polished, cleaned, and a heater/sensor element was placed between them. Full details of this process have been reported [28, 29]. The applied output power to the hot disk heater/sensor was 15 mW and the measurement time was 5 s. At least five tests per sample were carried out.

## 3 Results and discussion

### 3.1 Characterisation of GNPs and composite injection samples

**Figure 1:**
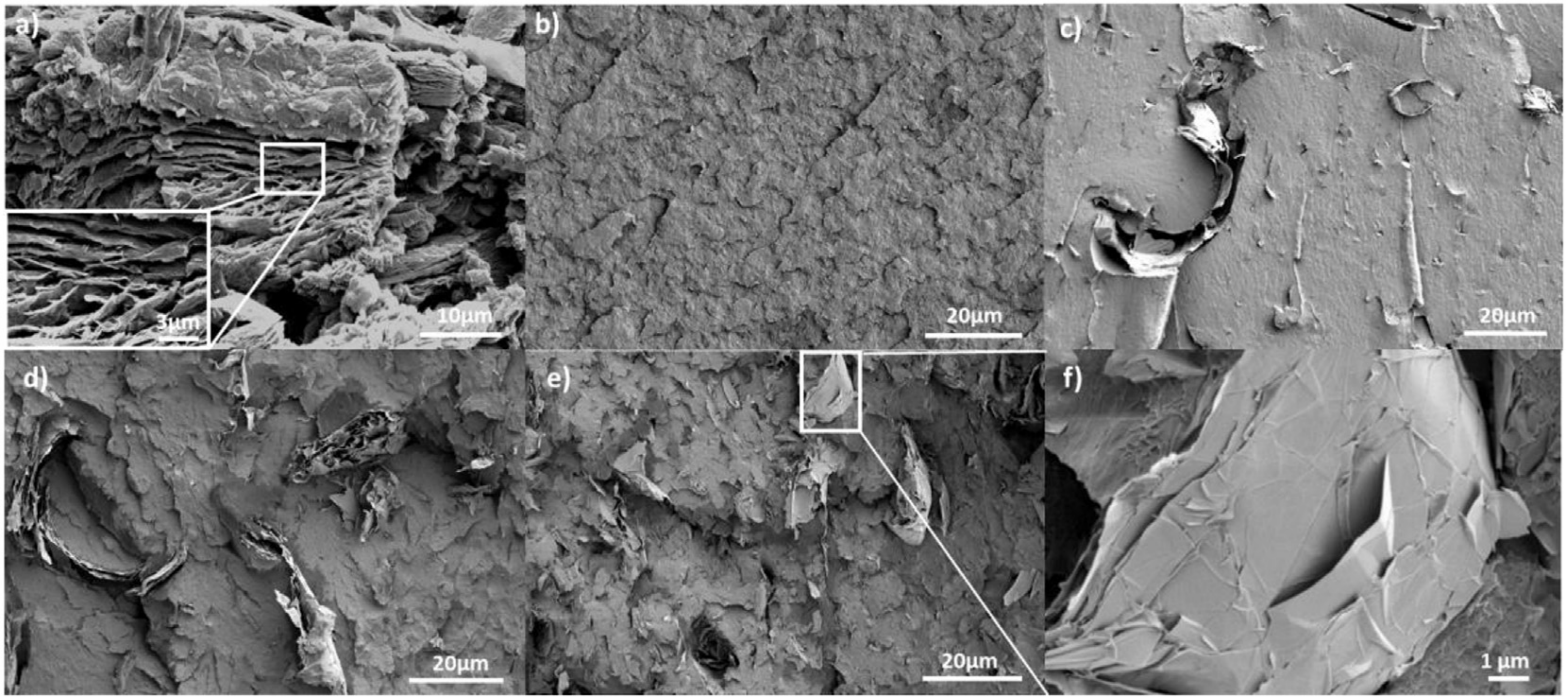
a), SEM images of as-received GNPs., SEM images of cryogenically fractured surfaces; b) neat PEEK, c) 1 wt.% GNP/PEEK specimen; d) 5 wt.% GNP/PEEK sample e) 10 wt.% GNP/PEEK sample, and f) SEM image of the magnified view of e) that shows agglomerates with numerous layers of graphene.

The morphology of the as-received graphene nanoplatelets was characterised by using SEM. Agglomerates of graphene are observed in Figure 1a. The SEM analysis showed a large distribution of sizes and number of layers of the agglomerates. No single or few layers of graphene were observed. The fractured surface of pure PEEK showed brittle behaviour, as expected (Figure 1b).

For the composite samples, the addition of GNP within the matrix slightly increased the density of the material (Table 2). As expected, the highest density was obtained for the 10 wt.% PEEK/GNP samples (1.332 ± 0.002 g/cm^3^), 3.3% more dense than pure PEEK. For all the samples, the differences among theoretical and the experimental density measures were lower than 1%, which indicates that the injection method works well, and the samples have low porosity. This low porosity matches the results of the SEM analysis and voids.

**Table 2:** Theoretical and experimental density values of PEEK/GNP composites.

Sample	Experimental density (g/cm3)	Theoretical density (g/cm3)
PEEK	1.288 ± 0.002	–
1 wt.% GNP	1.292 ± 0.003	1.305
5 wt.% GNP	1.313 ± 0.004	1.327
10 wt.% GNP	1.332 ± 0.001	1.355

With regards to nanofiller dispersion, for low loading of GNP (1 wt.%), we observed GNPs composed of a few stacks of graphene layers and good distribution of the GNP within the matrix (Figure 1c). For high loading of GNP (5 and 10 wt.%), bigger GNP agglomerates (compared to 1 wt.%) were observed (Figure 1d, e, f). In both cases, the size and content of the layers of graphene were significantly lower than the as-received GNPs (Figure 1a), which may indicate that exfoliation occurs during processing. Melt-compounding by extrusion and injection promotes better dispersion of GNP as a result of the intense shear stress present during processing, which promotes exfoliation of GNP sheets [24, 30]. The PEEK matrix penetrates the GNP agglomerates and separates the physically connected nano-platelets [30].

At the interface region between the nano reinforcement and the matrix, the PEEK completely wets the surface of most of the graphene agglomerates. However, debonded regions can be found among sheets of graphene, which can result from poor interactions between the graphene agglomerates and the PEEK or among the layers of graphene within agglomerates [21, 22] (Figure 1d). We did not functionalise the surface of graphene, which could improve graphene/PEEK adhesion [31].

### 3.2 Differential scanning calorimetry

The thermal properties of the injected samples were analysed by DSC. The degree of crystallinity (χ_c_) of the composites was calculated with the following equation [26]:

(1) $${\text{χ}}_{c}=\;\frac{\Delta H_{s}}{\left(1-\phi \right)\Delta H_{0}}$$

In which ΔH_S_ is the heat of melting calculated by integration from 130 °C (previous cold crystallisation) to 375 °C, ΔH_0_ is the heat of fusion of 100% crystalline PEEK, taken as 130 J/g [32] and ϕ is the weight fraction per unit mass of nanofiller.

**Figure 2:**
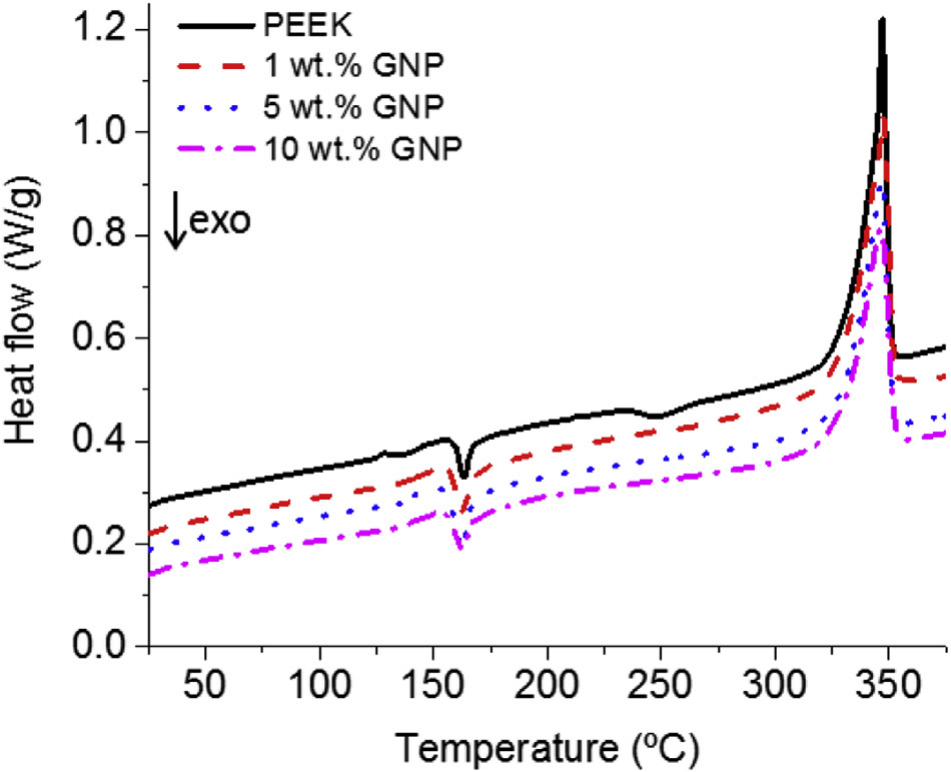
DSC thermographs that show heating curves of GNP/PEEK composites from dog-bone specimens.

From the first heating and later cooling ramps, both at 10 °C/min, thermal parameters such as glass transition, melting temperature, melting enthalpy, crystallinity, crystallisation temperature, and heat of crystallisation were calculated. Figure 2 shows the first heating ramp, in which the glass transition and melting temperature were not affected by adding GNP and remained constant at 141 °C and 346 °C. In all cases small cold crystallisation peaks are observed after the glass transition temperature [3]. This cold crystallisation is a consequence of the fast cooling during processing. A slight decrease in the temperature of the crystallisation peak, that is, the enthalpy of the cold crystallisation process is observed as the GNP concentration increases (Table 3), because GNP can accelerate the kinetic crystallisation of PEEK. This fact has been analysed through DSC and synchrotron radiation in our last work [33]. This lower cold crystallisation affects the crystallinity of the injected samples, which increases from 22.5% for pure PEEK to 26.2% for 10 wt.% PEEK/GNP (Table 3). This improvement in crystallinity is relative small, however it could affect the properties of the material because lower mechanical properties or lower environmental resistance have been reported with lower levels of crystallinity [34, 35].

**Table 3:** Thermal parameters of the samples. Crystallization (**T**_**c**_), melting (**T**_**m**_), and glass transition (**T**_**g**_) temperatures. Degree of crystallinity (**X**_**c**_) and the heat of melting calculated by integration from 130 °C (previous cold crystallization) to 375 °C (**ΔH**_**s**_). Heat of crystallization after melting (**ΔH**_**c**_).

Sample	T_c_ (°C)	T_m_ (°C)	T_g_ (°C)	ΔH_s_ (J/g)	X_c_ (%)	ΔH_c_ (J/g)
PEEK	307.0	346.2	140.8	29.26	22.5	53.3
1 wt.% GNP	307.8	346.8	140.5	31.61	24.6	50.2
5 wt.% GNP	309.9	346.9	140.2	28.92	23.4	48.3
10 wt.% GNP	311.5	346.6	140.9	30.71	26.2	47.5

### 3.3 Mechanical properties

The effect of GNPs on the mechanical properties of the composites has been explored by three-point bending and tensile tests, the results of which are shown in Table 4.

**Table 4:** Tensile and flexural properties of neat PEEK and PEEK/GNP composites.

Sample	Flexural test			Tensile test			
	E (GPa)	Strength (MPa)	Strain at break (mm/mm)	E (GPa)	Strength (MPa)	Strain (%)	Poisson's ratio
PEEK	3.52 ± 0.02	150.4 ± 1.1	13 ± 2	3.30 ± 0.05	93.8 ± 1.1	14.6 ± 1.4	0.38 ± 0.01
1 wt.% GNP	3.63 ± 0.05	150 ± 2	8.5 ± 0.3	3.47 ± 0.12	89 ± 2.0	8.1 ± 0.9	0.37 ± 0.01
5 wt.% GNP	3.75 ± 0.01	140.9 ± 1.3	6.2 ± 0.3	3.47 ± 0.03	84.7 ± 0.7	5.2 ± 0.2	0.35 ± 0.01
10 wt.% GNP	3.90 ± 0.03	128.2 ± 1.5	4.5 ± 0.1	4.08 ± 0.2	82 ± 3	3.6 ± 0.3	0.34 ± 0.01

**Figure 3:**
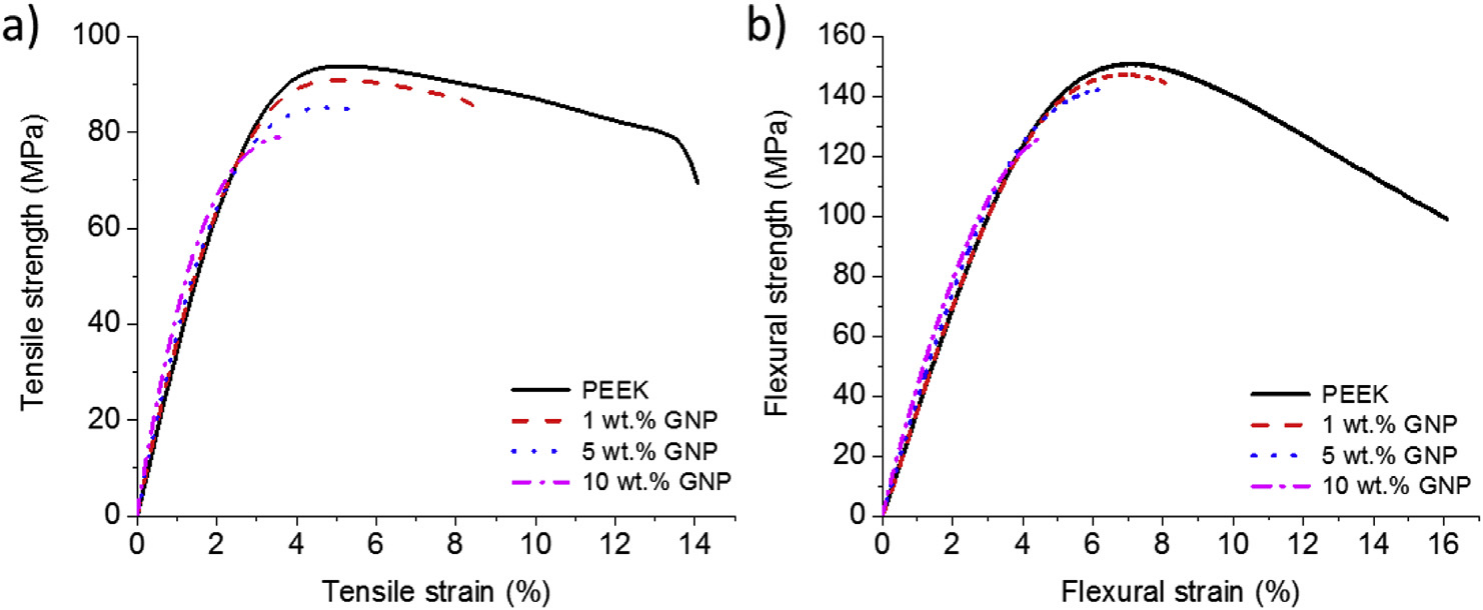
Examples of a) tensile curve and b) flexural curve for PEEK/GNP composites.

The addition of graphene increases the stiffness of the nanocomposite (see Figure 3). The nanocomposite with 10 wt.% GNP showed a 11% and 24% increase in flexural and tensile modulus, respectively, relative to baseline PEEK. The increase in tensile modulus with increase in GNP content [21, 22] may be a consequence of an increased interfacial area between the matrix and nanoparticles [4]. Similar improvements in PEEK/GNP composite modulus have been reported in melt processing (21% for 10 wt.% GNP) [22] and for solution processing (19% for 5 wt.% GNP) [21]. By following the semi-industrial melt-compounding approach with other PEEK and GNP composites, similar improvements in modulus (3%) and decreases in strength (7%) have been obtained for 1% wt. GNP/PEEK [36].

Conversely, the tensile and flexural strength slightly decreased with the addition of GNP. PEEK with 5 wt.% GNP exhibited an approximately 7% and 10% decrease in flexural and tensile strength, respectively. This reduction of strength can be attributed to a range of factors. Strong interfacial adhesion between the reinforcement and the matrix is essential to reach high strength in composites [18, 37]. In our case, because the graphene surface is not treated, the interaction between graphene and PEEK matrix may be poor and the load reinforcement-matrix transfer may be insufficient to achieve the strength of the nano-reinforcement under loading, thus decreasing the strength of the nanocomposite (Figure 3). Another reason for reduced strength might be agglomeration of GNPs within the matrix (see Figure 1) and weak interactions between the sheets of graphene [18]. Different behaviours about the strength of PEEK/GNP and other PEEK nanocomposites have been reported in the literature. Decreases of 9% and 3% in flexural and tensile strength, respectively, by the addition of 5 wt.% of GNP to PEEK dispersed by solution intercalation [4] and melt-compounding [8] have been reported. However, other studies have reported that the strength remained constant [7, 8, 22, 23] with addition of GNP or hydroxyapatite particles. Furthermore, the nanocomposite has lower strain at the breaking point (8% for 1% wt.% GNP) than pure PEEK samples (14%) (Figure 3), which might be related to poor PEEK/GNP interactions. This behaviour was previously reported in GNP and other nanofiller composites [7, 8, 21]. Nevertheless, an increase in strain can be achieved by improving the dispersion and the adhesion between the nanofiller and matrix. Rinaldi et al. [38], reported an increase in Young's modulus, strength and strain adding CNTs, owing to a good distribution and adhesion between the nanofiller and PEEK.

We also calculated Poisson's ratio (ν), which is a measure of the transverse contraction ($\varepsilon_{2})$, over the axial deformation ($\varepsilon_{1})$, both measured by DIC under a given axial stress σ_1_. The standard ν is defined as:

(2) $$\nu =\;\frac{\varepsilon_{2}\left(\sigma_{1}\right)}{\varepsilon_{1}\left(\sigma_{1}\right)}$$

The values reported in Table 4 are average Poisson's ratios for all data obtained during tensile tests. The average Poisson's ratio of pure PEEK was 0.375, which is close to the value of 0.38 that has been reported [39] by using ultrasonic sound speed technique.

When the content of graphene was low (1 wt.%), the Poisson's ratio was similar to pure PEEK, as expected [40]. But, the Poisson's ratio decreased by 7 and 9% with the addition of 5 and 10 wt.% of GNP, respectively, which indicates that the nanocomposite had lower transverse contraction than pure PEEK. This improvement was expected because GNPs have a higher Poisson's ratio (0.17) than that of the PEEK matrix [41, 42].

**Figure 4:**
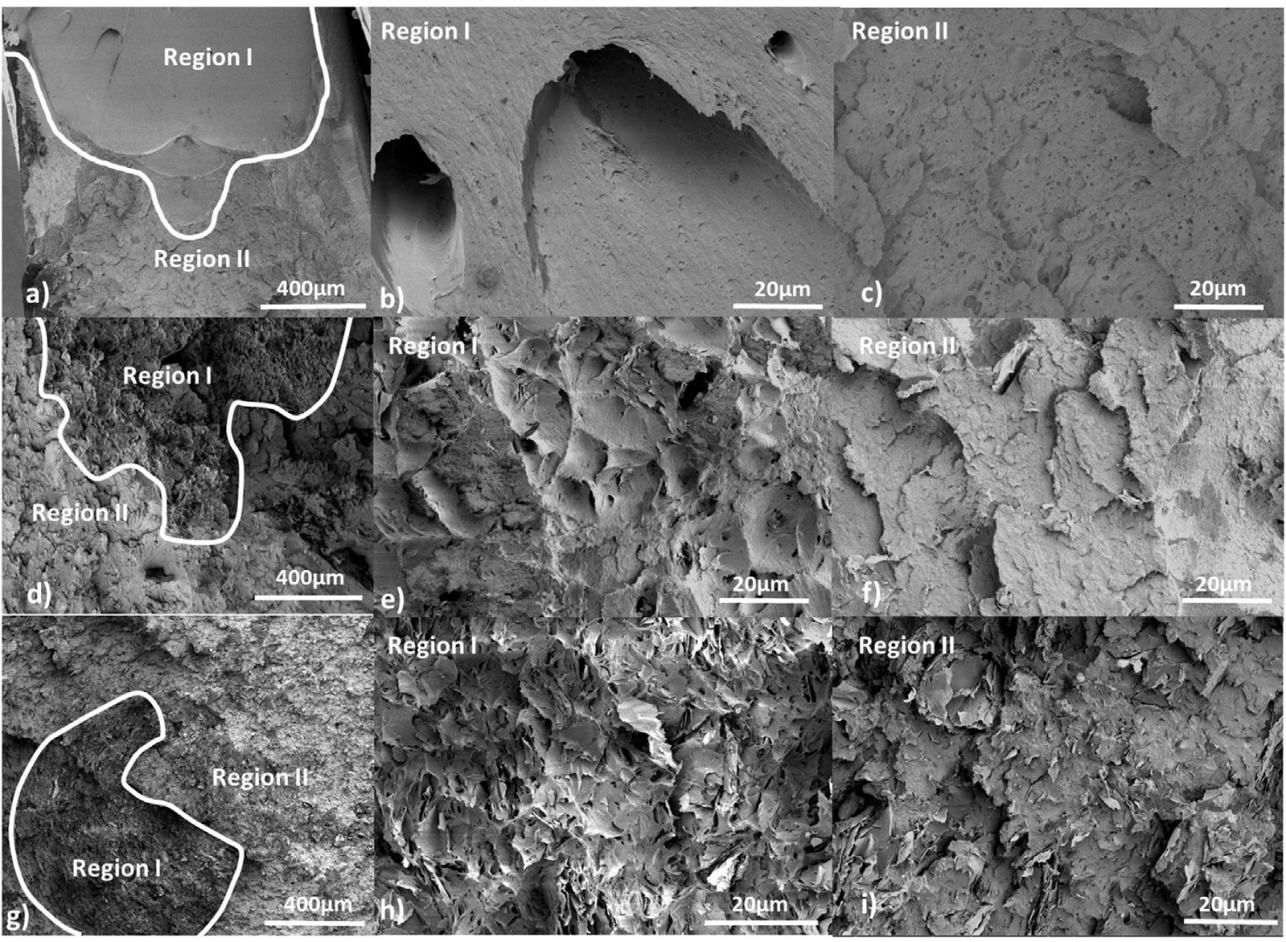
SEM images of fractured surfaces after tensile testing. a) Overview of pure PEEK surface; b) Region I, which corresponds to ductile behaviour; c) Region II, which corresponds to brittle behaviour in pure PEEK; d) Overview of 1 wt.% GNP/PEEK surface; e) Region I; and f) Region II in 1 wt.% GNP/PEEK sample: g) Overview of 10 wt.% GNP/PEEK surface: h) Region I; and i) Region II.

Fractography analysis of the broken surfaces of pure PEEK showed two different regions; Region I and Region II [7] (Figure 4a). Figure 4b shows Region I, which corresponds to fracture initiation. The fracture morphology observed in this region suggests ductile deformation rather than stable crack growth. More than one critical crack initiation, which forms a parabolic pattern, can be seen [43]. Voids and crazes that were formed in region I produce long-term “fibration”, which can be seen in Figure 4b [7]. Figure 4c shows the morphology of Region II (fracture propagation region). In this case, the morphology suggests fast and brittle fracture of pure PEEK.

For the nanocomposites, two distinct regions can be seen (Figure 4d and g). In the case of 1 and 10 wt.% PEEK/GNP samples, Region II (brittle behaviour) was similar to Region II for pure PEEK (Figure 4f, i). Nevertheless, the morphology of Region I was different from that of pure PEEK. In Figure 4e and h, there are numerous dimples in this region, which have been observed by other researchers with the addition of other fillers, such as PEEK/SiO_2_ nanocomposites [7]. GNP might produce stress concentration sites in a matrix and act as a crack nucleation site that promotes the formation of dimples. When the content of GNP was increased, the size of Region I, which is related with the high plastic deformation failure of the matrix, decreased and almost disappeared in the case of 10 wt.% of GNP. This explains the decrease of matrix ductility observed by the addition of GNP (Figure 3).

In addition, with increasing GNP content, the size of the dimples decreases as a result of the formation of larger number of nanoplates per volume fraction of material (Figure 4e, h). However, GNP can block the ductile flow of the matrix, which results in enhanced stiffness of the nanocomposites with respect to pure PEEK.

### 3.4 Thermal conductivity

**Figure 5:**
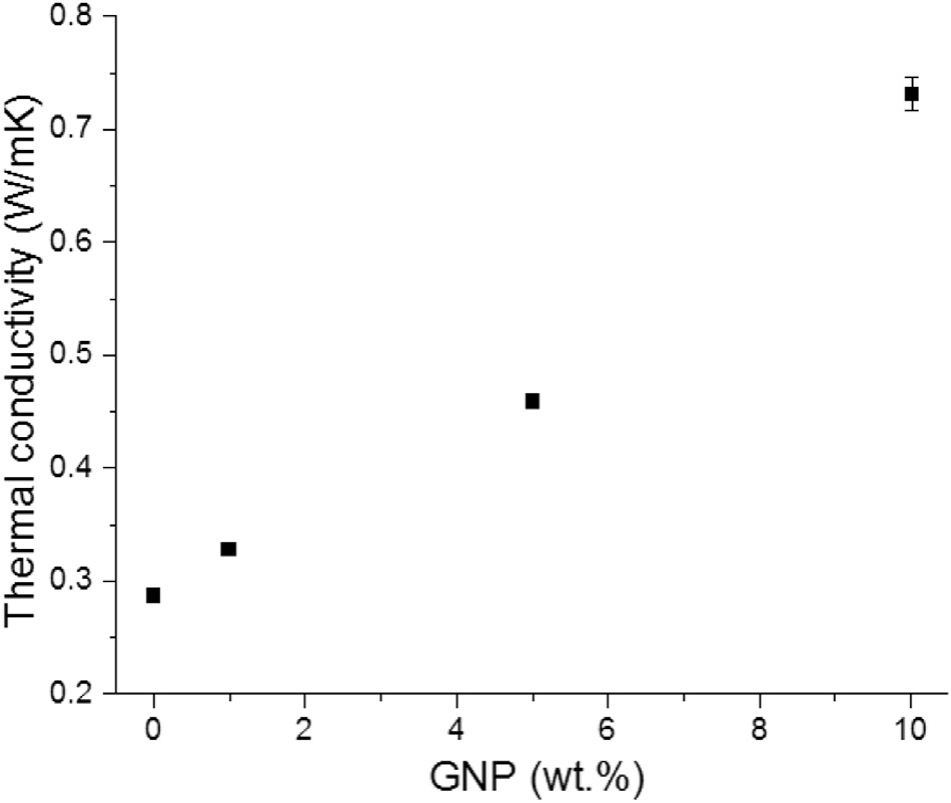
Variation of thermal conductivity with content of graphene.

The thermal conductivity of PEEK/GNP composites versus weight fraction of GNP is shown in Figure 5. The thermal conductivity of pure PEEK was 0.289 ± 0.003 W/mK, which was in agreement with the supplier datasheet. The values of the composites showed a linear increase as the fraction of GNP increased. The PEEK sample with 1 wt.% GNP has a thermal conductivity of 0.329 ± 0.002 W/mK, whereas for PEEK with 10 wt.% GNP, the thermal conductivity increases to 0.73 ± 0.01 W/mK, which is more than double the value obtained for pure PEEK. This behaviour change can be attributed to the high thermal conductivity of GNP [21, 44]. The PEEK/GNP nanocomposites with high GNP content were seen to have larger agglomerates (Figure 1) with many connections between them, which favours thermal conductivity.

This value (0.73 ± 0.01 W/mK for 10 wt.% GNP, 120% improvement) is the highest thermal conductivity measured and reported for PEEK/GNP composites so far; note that 37% and 90% improvements have been reported [22, 45] for similarly manufactured materials with 10% of GNP and 10% of multi-walled carbon nanotubes, respectively. Comprehensive research is now needed to understand the thermal conductivity behaviour of these samples.

However, the enhancement of 10 wt.% of GNP was moderate relative to the previous results with other polymeric matrices or similar nanofillers. As an example, Hwang et al. [5] reported an enhancement of 100% by adding 1 wt.% of graphene oxide (GO). Therefore, higher conductivities could be achieved by enhancing the processing to obtain better GNP dispersion and distribution. The number of layers and size of GNP are key to maximise the thermal conductivity enhancement of the nanocomposites and obtain the best result by adding a single or bi-layer graphene [44].

## 4 Conclusions

High performance PEEK/GNP nanocomposite samples even with high GNP loadings (5 and 10 wt.%) could be manufactured by an industrial two-step process (melt-compounding and injection moulding). This study shows how current industrial machines can be used to manufacture nanocomposite parts in the industry.

The processing conditions were enough to obtain good dispersion of GNP agglomerates at low concentrations (1 wt.%). However, with increased amounts of GNP, larger agglomerates of GNP were found. The size of the agglomerates, even at 10 wt.% GNP, is smaller than in pristine GNP, which shows how efficient this processing technique is at GNP dispersion. These GNP/PEEK composites crystallise faster than pure PEEK, probably because GNP has a nucleating effect that results in higher crystallinities after crystallisation at high cooling rates.

The addition of GNP within the PEEK matrix enhances the elastic modulus of PEEK, but decreases the strength, which indicates that there is poor transfer between nano-reinforcement and matrix. Furthermore, graphene decreases the Poisson's ratio of pure PEEK. Different failure behaviours were observed by fractography of the nanocomposite samples, which were probably a result of a decrease in strain at break through the addition of GNP. Higher contents of GNP showed larger areas associated with brittle behaviour.

The introduction of graphene to the PEEK matrix improved the thermal conductivity. The sample with 10 wt.% GNP exhibited 0.73 W/mK, which is two-fold the thermal conductivity of pure PEEK. This significant increase has never observed for GNP nanocomposites manufactured by melt-compounding.

To improve the dispersion and enhance the stress transfer across the interface between PEEK and graphene, a change in the semi-industrial parameters and the use of complementary techniques, such as functionalisation of graphene, or the use of compatibilizer agents need to be explored. To conclude, we have demonstrated how a semi-industrial technique can produce PEEK nanocomposites with enhanced thermal properties with retained or even slightly improved mechanical properties.

## Declarations

### Author contribution statement

A. Alvaredo-Atienza: Performed the experiments; Analyzed and interpreted the data; Wrote the paper.

Juan P. Fernández-Blázquez: Analyzed and interpreted the data.

P. Castell: Performed the experiments; Contributed reagents, materials, analysis tools or data.

Guzmán de Villoria: Conceived and designed the experiments; Analyzed and interpreted the data; Contributed reagents, materials, analysis tools or data; Wrote the paper.

### Funding statement

This work was supported by the 10.13039/100012818Community of Madrid through the NMAT2D-CM (S2018/NMT-4511). R. Guzman de Villoria was supported by the Spanish 10.13039/501100003176Ministry of Education, Culture and Sports through the Beatriz Galindo fellowship (BEAGAL18/00091).

### Competing interest statement

The authors declare no conflict of interest.

### Additional information

No additional information is available for this paper.
